# Impact of Severe Postoperative Multi‐Organ Dysfunction on Long‐Term Outcomes After Modified Morrow Surgery

**DOI:** 10.1002/clc.70434

**Published:** 2026-08-03

**Authors:** Tong Wang, La Ta, Kui Zhang, Ran Dong, Yu Xiao, Jiayang Wang

**Affiliations:** ^1^ Institute of Cardiac Surgery Beijing An Zhen Hospital Capital Medical University Beijing China

**Keywords:** hypertrophic obstructive cardiomyopathy, long‐term outcomes, major adverse cardiac and cerebrovascular events, modified morrow surgery, multi‐organ dysfunction, SOFA score

## Abstract

**Background:**

The modified Morrow surgery is the gold standard surgical treatment for hypertrophic obstructive cardiomyopathy (HOCM). While effective, it carries a risk of postoperative severe multi‐organ dysfunction and its impact on long‐term cardiovascular outcomes remains poorly understood and controversial.

**Methods:**

This observational cohort study included 380 HOCM patients undergoing the modified Morrow surgery between July 2015 and March 2025. Severe multi‐organ dysfunction was defined as a maximum Sequential Organ Failure Assessment (SOFA) score ≥ 11. The primary endpoint was the long‐term incidence of major adverse cardiac and cerebrovascular events (MACCEs). Secondary endpoints included heart failure hospitalization (HFH), postoperative atrial fibrillation (AF) ablation, and cardiovascular mortality.

**Results:**

Compared to patients without severe multi‐organ dysfunction (SOFA Maximum < 11, *n* = 234), those with SOFA Maximum ≥ 11 (*n* = 146) had significantly higher rates of MACCEs (53.4% vs. 17.5%, *p* < 0.001), cardiovascular mortality (11.6% vs. 0.9%, *p* < 0.001), HFH (21.9% vs. 8.5%, *p* < 0.001), and postoperative AF ablation (17.8% vs. 3.8%, *p* < 0.001). Multivariable Cox proportional hazards regression demonstrated that severe multi‐organ dysfunction was independently associated with cardiovascular mortality (HR = 4.157, *p* = 0.047), MACCEs (HR = 2.296, *p* < 0.001), HFH (HR = 1.834, *p* = 0.042), and postoperative AF ablation (HR = 3.987, *p* < 0.001). Other independent predictors included family history of hypertrophic cardiomyopathy, left ventricular end‐systolic dimension, postoperative left ventricular outflow tract velocity, and postoperative surgical intensive care unit ventilation time.

**Conclusions:**

Postoperative severe multi‐organ dysfunction is independently associated with an increased risk of adverse long‐term outcomes after modified Morrow surgery. Incorporating the SOFA score into postoperative risk stratification may help identify high‐risk patients warranting intensified long‐term management.

## Graphical abstract

This retrospective cohort study (*n* = 380) demonstrates that severe postoperative multi‐organ dysfunction (SOFA Maximum ≥ 11) is a strong independent predictor of long‐term adverse outcomes after modified Morrow surgery. Incorporating the SOFA score into postoperative assessments helps identify high‐risk hypertrophic obstructive cardiomyopathy patients warranting heightened clinical vigilance.

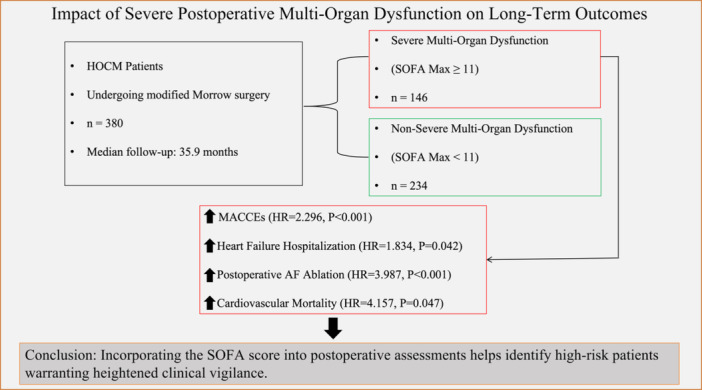

**Abbreviations**
AF: atrial fibrillation
AUC: area under the curve
BMI: body mass index
BNP: B‐type natriuretic peptide
CI: confidence intervals
CPB: cardiopulmonary bypass.
HCM: hypertrophic cardiomyopathy
HFH: heart failure hospitalization
HOCM: hypertrophic obstructive cardiomyopathy
HR: hazard ratio
IVST: interventricular septal thickness
LAD: left atrial diameter
LDH: lactate dehydrogenase
LVEDD: left ventricular end‐diastolic dimension
LVEF: left ventricular ejection fraction
LVESD: left ventricular end‐systolic dimension
LVOT: left ventricular outflow tract
MACCEs: major adverse cardiac and cerebrovascular events
MI: myocardial infarction
MR: mitral regurgitation
NSVT: non‐sustained ventricular tachycardia
OR: odds ratios
Post‐SICU Vent Time: postoperative surgical intensive care unit ventilation time
PPM: postoperative permanent pacemaker
ROC: receiver operating characteristic
SAM: systolic anterior motion of the mitral valve
SCD: sudden cardiac death
SICU: surgical intensive care unit
SOFA: Sequential Organ Failure Assessment

## 1 Introduction

As the gold standard surgical treatment for hypertrophic obstructive cardiomyopathy (HOCM), the modified Morrow surgery alleviates left ventricular outflow tract (LVOT) obstruction by resecting hypertrophied septal myocardium, thereby significantly reducing LVOT gradient and improving patients' cardiac function and clinical symptoms [1, 2, 3, 4]. Despite continuous advancements in surgical techniques, there remains a certain perioperative risk, among which the incidence of severe multi‐organ dysfunction is relatively high, making it one of the main causes of early postoperative mortality [5, 6, 7]. Furthermore, patients undergoing this procedure also face a high risk of long‐term major adverse cardiac and cerebrovascular events (MACCEs), such as cardiovascular death, atrial fibrillation (AF)‐related stroke, various arrhythmias, and the need for postoperative permanent pacemaker (PPM) implantation, etc [2, 6, 8, 9].

However, a critical and unresolved controversy persists in clinical practice: whether surviving an episode of severe multi‐organ dysfunction represents a reversible “hit” with long‐term outcomes comparable to those of uncomplicated patients, or if it induces irreversible damage that portends a persistently elevated risk of adverse cardiovascular events. This controversy is exacerbated by the silence of current clinical guidelines and expert consensus documents, which provide detailed recommendations for the acute management of severe multi‐organ dysfunction but offer no guidance on the long‐term stratification and follow‐up for this unique survivor population. Existing studies predominantly focus on the prevention and management of in‐hospital mortality and short‐term complications, yet fail to thoroughly investigate the association between severe multi‐organ dysfunction after cardiac surgery and long‐term outcomes (such as cardiovascular mortality, heart failure hospitalization (HFH), quality of life, and composite cardiovascular endpoints) [10, 11]. Some studies suggest that the impact of severe multi‐organ dysfunction on long‐term prognosis is reversible, and this process recovers as the patient recovers [12]. In contrast, other studies indicate that it may cause irreversible organ damage, becoming an “initiating factor” for long‐term adverse prognosis [13, 14]. Therefore, clarifying the relationship between severe multi‐organ dysfunction after modified Morrow surgery and long‐term prognosis not only provides objective evidence for understanding their correlation, but also holds significant clinical implications for improving postoperative risk stratification and optimizing long‐term follow‐up strategies.

The present study aims to systematically evaluate the impact of severe multi‐organ dysfunction on long‐term survival and cardiac functional outcomes following the modified Morrow surgery through medium‐ to long‐term follow‐up. The findings will provide clinicians with valuable prognostic tools, facilitate the identification of high‐risk populations, and thereby enable the implementation of more aggressive perioperative interventions to improve patients' long‐term outcomes.

## 2 Methods

### 2.1 Study Population

This observational cohort study included 380 patients diagnosed with HOCM who underwent the modified Morrow surgery at Beijing Anzhen Hospital from July 2015 to March 2025. Inclusion criteria were as follows: (1) patients aged over 18 years; (2) diagnosis of HOCM confirmed by imaging techniques such as echocar­diography and cardiac magnetic resonance imaging; (3) patients who underwent modified Morrow surgery; and (4) availability of complete preoperative and postoperative follow‐up data. Exclusion criteria included: (1) presence of other severe cardiovascular diseases; (2) preoperative severe liver or kidney dysfunction or infection; and (3) loss to follow‐up or incomplete follow‐up data after procedure.

Follow‐up was conducted annually through clinical visits, telephone interviews, and review of medical records. The follow‐up time was defined as the period from surgery to either the date of the last contact (if no event occurred) or the date of the event. The median follow‐up time was 35.90 months (interquartile range [IQR]: 20.20−47.02 months). The Research Ethics Committee of Beijing Anzhen Hospital approved the study (approval number: KS2025079), and written informed consent was waived for all patients. The study conformed to the principles outlined in the Declaration of Helsinki and was conducted under the guide‐lines of the Institutional Review Board.

### 2.2 Data Collection

Data were collected from all patients' preoperative assessments, intraoperative records, and postoperative clinical data (as shown in Table 1). The collected variables included:

**Table 1:** Patients' baseline characteristics.

Variable	Non‐severe multi‐organ dysfunction (SOFA maximum< 11) (*n* = 234)	Severe multi‐organ dysfunction (SOFA maximum ≥ 11) (*n* = 146)	*p* value
Male, *n* (%)	138 (59.0%)	75 (51.4%)	0.146
Age, years	53 (43, 61)	59 (52, 66)	**<0.001**
BMI, kg/m^2^	26.025 (23.8875, 28.2275)	25.96 (23.3625, 28.05)	0.266
Family history of HCM, *n* (%)	51 (21.8%)	55 (37.7%)	**0.001**
Family history of SCD, *n* (%)	37 (15.8%)	34 (23.3%)	0.069
Unexplained Syncope, *n* (%)	55 (23.5%)	50 (34.2%)	**0.023**
Chest discomfort, *n* (%)	222 (94.9%)	136 (93.2%)	0.485
Diabetes mellitus, *n* (%)	18 (7.7%)	15 (10.3%)	0.385
Hypertension, *n* (%)	85 (36.3%)	75 (51.4%)	**0.004**
Hypercholesterolemia, *n* (%)	21 (9.0%)	23 (15.8%)	**0.045**
Coronary artery disease, *n* (%)	25 (10.7%)	26 (17.8%)	**0.048**
LAD, mm	41 (38, 46)	42.2 (39.3, 46)	0.159
LVESD, mm	27.498 ± 4.1067	27.292 ± 4.3075	0.640
LVEDD, mm	43.498 ± 4.947	42.698 ± 4.6973	0.119
Moderate/Severe MR, *n* (%)	180 (76.9%)	110 (75.3%)	0.724
LVOTG, mmHg	72.5 (51, 97.85)	67.3 (50.75, 97)	0.576
LVOTV, cm/s	422.1 ± 113.57	413.23 ± 118.75	0.467
SAM, *n* (%)	221 (94.4%)	124 (84.9%)	**0.002**
Post‐LVOTV, cm/s	170.9 (129.5, 212.25)	169.5 (140.45, 210.15)	0.879
Post‐LVOTG, mmHg	12.8 (8, 21)	12 (8, 20.7)	0.720
LVPWT, mm	12 (10.615, 13.65)	12.7 (11, 15)	0.199
IVST, mm	21 (19, 24)	21 (19, 24)	0.577
E/A	0.870 (0.7, 1.335)	0.855 (0.7, 1.1825)	0.391
LVEF, %	65 (60, 69)	62.8 (59, 68)	0.061
BNP, pg/mL	371 (184.75, 656.5)	580.5 (301, 987.8)	**<0.001**
CK‐MB, ng/mL	2.5 (1.8, 3.425)	2.5 (1.8, 3.4)	0.868
LDH, U/L	207.7 (179, 232.85)	216 (183, 248.5)	**0.036**
NSVT, *n* (%)	48 (20.5%)	35 (24.0%)	0.427
Surg Time, h	5.355 (4.8225, 6.17)	6 (5, 7.0725)	**<0.001**
Post‐SICU vent time, h	21 (17, 26)	26 (18.5, 49.625)	**<0.001**

*Note:* Bold values indicate statistical significance (*p* < 0.05).

Abbreviations: BMI = body mass index, BNP = B‐type natriuretic peptide, CK‐MB = creatine kinase‐myocardial band, E/A = early to late diastolic mitral inflow velocity ratio, HCM = hypertrophic cardiomyopathy, IVST = interventricular septal thickness, LDH = lactate dehydrogenase, NSVT = non‐sustained ventricular tachycardia, LAD = left atrial diameter, LVEDD = left ventricular end‐diastolic dimension, LVEF = left ventricular ejection fraction, LVESD = left ventricular end‐systolic dimension, LVOTG = left ventricular outflow tract gradient, LVOTV = left ventricular outflow tract velocity, LVPWT = left ventricular posterior wall thickness, Moderate/Severe MR = moderate to severe mitral regurgitation, Post‐LVOTG = postoperative left ventricular outflow tract gradient, Post‐LVOTV = Postoperative left ventricular outflow tract velocity, Post‐SICU Vent Time = postoperative surgical intensive care unit ventilation time, SAM = systolic anterior motion of the mitral valve, SCD = sudden cardiac death, SOFA = Sequential Organ Failure Assessment, Surg Time = surgery time.

#### 2.2.1 Demographics and Baseline Characteristics

Male, age, body mass index (BMI), family history (including family history of hypertrophic cardiomyopathy [HCM] and sudden cardiac death [SCD]), clinical symptoms (chest discomfort, unexplained syncope), and comorbidities (hypertension, hypercholesterolemia, diabetes mellitus, coronary artery disease).

#### 2.2.2 Echocardiographic Parameters

Left atrial diameter (LAD), left ventricular end‐systolic dimension (LVESD), left ventricular end‐diastolic dimension (LVEDD), moderate/severe mitral regurgitation (MR), LVOT gradient, LVOT velocity, systolic anterior motion of the mitral valve (SAM), postoperative LVOT gradient, postoperative LVOT velocity, left ventricular posterior wall thickness, interventricular septal thickness (IVST), early to late diastolic mitral inflow velocity ratio, and left ventricular ejection fraction (LVEF).

#### 2.2.3 Laboratory Biomarkers

B‐type natriuretic peptide (BNP), creatine kinase‐myocardial band, and lactate dehydrogenase (LDH).

#### 2.2.4 Electrocardiographic Findings

Non‐sustained ventricular tachycardia (NSVT).

#### 2.2.5 Intraoperative Variables and Outcome Data

Surgery time and postoperative surgical intensive care unit ventilation time (Post‐SICU Vent Time).

### 2.3 Definitions and Study Endpoints

**Figure 1:**
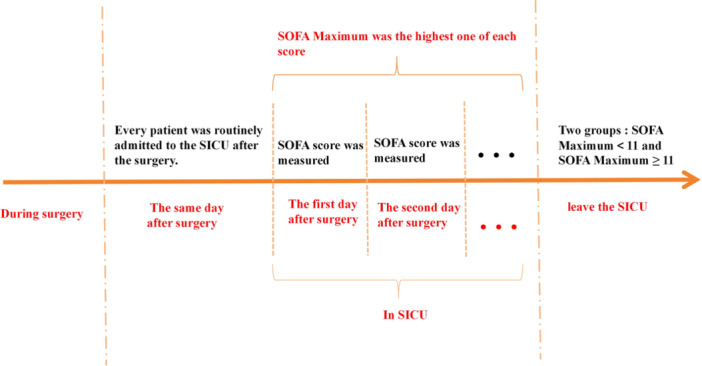
The point‐in‐time of measurement of SOFA score. SICU = surgical intensive care unit, SOFA = Sequential Organ Failure Assessment.

**Figure 2:**
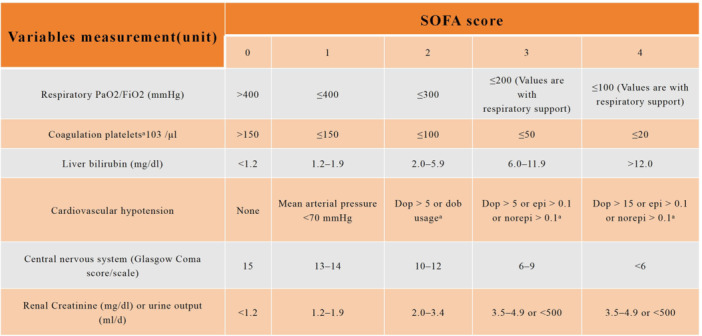
The definition of SOFA score. Dob = dobutamine, Dop = dopamine, Epi = epinephrine, FiO2 = fraction of inspired oxygen, Norepi = norepinephrine, SOFA = Sequential Organ Failure Assessment. ^a^Adrenergic agents administered for at least 1 h (doses given are in ug/kg per min).

Studies have demonstrated that the SOFA score serves as the gold standard for prognostic evaluation after cardiac surgery, and the score itself can function as an independent predictor of in‐hospital mortality following cardiac surgical procedures [14, 15, 16]. Prior studies have also recommended utilizing the maximum SOFA score to evaluate progressive multi‐organ dysfunction, with a maximum SOFA score ≥ 11 predicting a 95% mortality rate following inpatient cardiac surgery [17, 18]. Therefore, we defined a maximum SOFA score ≥ 11 as indicative of severe multi‐organ dysfunction following modified Morrow surgery. The maximum SOFA score was determined by daily assessments starting from the first postoperative day until discharge from the surgical intensive care unit (SICU), with the highest recorded value designated as the maximum SOFA score. The timing of SOFA score measurements is illustrated in Figure 1. Scores were calculated based on vital signs, laboratory results, and medication records from the SICU electronic medical records, and were independently assessed by two researchers blinded to patients' long‐term outcomes. The criteria for SOFA score calculation are detailed in Figure 2.

The composite endpoint for long‐term follow‐up was defined as MACCEs, which served as the primary endpoint. MACCEs included SCD, cardiovascular mortality, AF‐related stroke, HFH, myocardial infarction(MI), postoperative PPM implantation, postoperative AF ablation, and postoperative AF. To more precisely investigate the relationship between severe multi‐organ dysfunction and specific long‐term adverse outcomes, we separately analyzed HFH, postoperative AF ablation, and cardiovascular mortality from the MACCEs components. These were designated as secondary endpoints. All endpoint events were independently adjudicated by two senior cardiologists who were blinded to patients' SOFA score groupings. Any disagreements were resolved through arbitration by a third expert.

### 2.4 Statistical Analysis

Continuous variables were evaluated for normal distribution using the Shapiro−Wilk test. Normally distributed continuous variables were presented as mean ± standard deviation and compared using the independent samples Student's *t*‐test. Non‐normally distributed variables were expressed as median with interquartile range and compared using the Mann−Whitney *U* test. Categorical variables were reported as frequencies with percentages, and group comparisons were performed using Pearson's chi‐square test or Fisher's exact test (strictly applied when the expected count in any cell was less than 5).

Because the primary and secondary endpoints were time‐to‐event outcomes with varying follow‐up durations, cumulative event rates were visualized using Kaplan‐Meier survival curves, and differences between groups were assessed utilizing the log‐rank test. To identify independent predictors for the endpoints during the follow‐up period, Cox proportional hazards regression models were constructed. Variables demonstrating a potential association (*p* < 0.05) in the univariate Cox regression analysis were evaluated for multicollinearity using the Variance Inflation Factor (VIF). Variables with a VIF < 5 were subsequently incorporated into the multivariable Cox proportional hazards model. The results were presented as hazard ratios (HR) with their corresponding 95% Confidence Intervals (CI). All statistical hypotheses were tested two‐sided, and a *p*‐value < 0.05 was considered statistically significant.

All statistical analyses were performed using SPSS version 25.0 (IBM Corporation, Armonk, NY, USA). Figures were created using GraphPad Prism version 10.5 (GraphPad Software, San Diego, CA, USA) and WPS Office version 12.1.22218 (Kingsoft Corporation, Beijing, China).

## 3 Results

### 3.1 Baseline Characteristics

A total of 380 patients with HOCM who underwent the modified Morrow surgery were enrolled and stratified into two cohorts based on the occurrence of severe postoperative multi‐organ dysfunction: a non‐severe group (SOFA Maximum < 11, *n* = 234) and a severe group (SOFA Maximum ≥ 11, *n* = 146). As detailed in Table 1, patients developing severe multi‐organ dysfunction were significantly older (median age 59 vs. 53 years, *p* < 0.001) and exhibited a higher baseline burden of cardiovascular comorbidities, including hypertension (51.4% vs. 36.3%, *p* = 0.004), hypercholesterolemia (15.8% vs. 9.0%, *p* = 0.045), and coronary artery disease (17.8% vs. 10.7%, *p* = 0.048). Furthermore, the severe dysfunction cohort had a significantly higher prevalence of a family history of HCM (37.7% vs. 21.8%, *p* = 0.001) and unexplained syncope (34.2% vs. 23.5%, *p* = 0.023), alongside a lower incidence of preoperative SAM (84.9% vs. 94.4%, *p* = 0.002). Perioperatively, these high‐risk patients presented with elevated baseline BNP levels [580.5 vs. 371 pg/mL, *p* < 0.001] and LDH levels [216 vs. 207.7 U/L, *p* = 0.036]. They also experienced significantly longer surgery times [6 vs. 5.355 h, *p* < 0.001] and prolonged post‐SICU Vent Time [26 vs. 21 h, *p* < 0.001]. Conversely, no statistically significant differences were observed between the two groups regarding sex, BMI, left ventricular structural dimensions (e.g., LVEDD, LVESD, and LAD), LVEF, baseline outflow tract gradients, or NSVT (all *p* > 0.05).

### 3.2 Long‐Term Follow‐Up Outcomes

In terms of long‐term follow‐up outcomes (Table 2), the severe multi‐organ dysfunction group had significantly higher rates of cardiovascular mortality (11.6% [*n* = 17] vs. 0.9% [*n* = 2], *p* < 0.001), HFH (21.9% [*n* = 32] vs. 8.5% [*n* = 20], *p* < 0.001), postoperative AF ablation (17.8% [*n* = 26] vs. 3.8% [*n* = 9], *p* < 0.001), and MACCEs composite endpoint incidence (53.4% [*n* = 78] vs. 17.5% [*n* = 41], *p* < 0.001) compared to the non‐severe multi‐organ dysfunction group.

**Table 2:** Long‐term follow‐up outcome baseline characteristics.

Outcome	Non‐severe multi‐organ dysfunction (SOFA maximum < 11) (*n* = 234)	Severe multi‐organ dysfunction (SOFA maximum ≥ 11) (*n* = 146)	*p* value
SCD, *n* (%)	2 (0.9%)	2 (1.4%)	0.640
Cardiovascular mortality, *n* (%)	2 (0.9%)	17 (11.6%)	< 0.001
HFH, *n* (%)	20 (8.5%)	32 (21.9%)	< 0.001
AF‐related stroke, *n* (%)	1 (0.4%)	5 (3.4%)	0.033
MI, *n* (%)	3 (1.3%)	6 (4.1%)	0.092
Postoperative PPM implantation, *n* (%)	3 (1.3%)	6 (4.1%)	0.092
Postoperative AF ablation, *n* (%)	9 (3.8%)	26 (17.8%)	< 0.001
Postoperative AF, *n* (%)	17 (7.3%)	21 (14.4%)	0.024
MACCEs, *n* (%)	41 (17.5%)	78 (53.4%)	< 0.001

Abbreviations: AF = atrial fibrillation, HFH = heart failure hospitalization, MACCEs = major adverse cardiac and cerebrovascular events, MI = myocardial infarction, PPM = permanent pacemaker, SCD = sudden cardiac death, SOFA = Sequential Organ Failure Assessment.

### 3.3 Univariate and Multivariable Cox Proportional Hazards Regression Analyses

Univariable Cox regression analyses were initially performed to identify potential risk factors associated with the primary and secondary endpoints. Severe multi‐organ dysfunction was significantly associated with all endpoints in the univariable analysis (all *p* < 0.05). Detailed results of the univariable analyses for all variables are provided in the Supporting Information Materials.

Multivariable Cox proportional hazards regression analysis was subsequently performed with the incidence of MACCEs, HFH, postoperative AF ablation, and cardiovascular mortality as dependent time‐to‐event variables. The results (Table 3) demonstrated that severe multi‐organ dysfunction was independently associated with increased risks of MACCEs (HR = 2.296, 95% CI: 1.528−3.486, *p* < 0.001), HFH (HR = 1.834, 95% CI: 1.023−3.352, *p* = 0.042), postoperative AF ablation (HR = 3.987, 95% CI: 1.885−9.181, *p* < 0.001), and cardiovascular mortality (HR = 4.157, 95% CI: 1.017−28.11, *p* = 0.047). Additionally, a family history of HCM was significantly associated with increased risks of cardiovascular mortality (HR = 10.58, *p* < 0.001) and HFH (HR = 2.079, *p* = 0.013). Increased post‐SICU Vent Time significantly elevated the cardiovascular mortality risk (HR = 1.004, *p* = 0.031). Furthermore, larger LVESD was significantly associated with an increased risk of MACCEs (HR = 1.048, *p* = 0.018), whereas higher Post‐LVOTV was associated with the risk of postoperative AF ablation (HR = 0.9924, *p* = 0.013).

**Table 3:** Multivariable Cox regression analysis for primary and secondary endpoints.

Endpoint	Variable	Multivariate analysis HR	95% CI	*p* value
MACCEs	Family history of HCM	1.191	0.8059 to 1.744	0.376
	LVESD, mm	1.048	1.008 to 1.090	**0.018**
	Surg time, h	1.102	0.9824 to 1.233	0.097
	Post‐SICU Vent Time, h	1.002	0.9995 to 1.003	0.133
	Severe multi‐organ dysfunction (SOFA maximum ≥ 11)	2.296	1.528 to 3.486	**<0.001**
HFH	Family history of HCM	2.079	1.172 to 3.689	**0.013**
	Severe multi‐organ dysfunction (SOFA maximum ≥ 11)	1.834	1.023 to 3.352	**0.042**
Postoperative AF ablation	Post‐LVOTV, cm/s	0.9924	0.9860 to 0.9985	**0.013**
	Surg time, h	1.192	0.9806 to 1.440	0.077
	Severe multi‐organ dysfunction (SOFA maximum ≥ 11)	3.987	1.885 to 9.181	**<0.001**
Cardiovascular mortality	Age, years	1.029	0.9877 to 1.077	0.174
	Family history of HCM	10.58	2.976 to 50.97	**<0.001**
	Family history of SCD	0.5806	0.1812 to 1.796	0.343
	LVEDD, mm	0.9601	0.8643 to 1.060	0.423
	Surg time, h	1.261	0.9667 to 1.639	0.086
	Post‐SICU vent time, h	1.004	1.000 to 1.008	**0.031**
	Severe multi‐organ dysfunction (SOFA maximum ≥ 11)	4.157	1.017 to 28.11	**0.047**

*Note:* Bold values indicate statistical significance (*p* < 0.05).

Abbreviations: AF = atrial fibrillation, CI = confidence interval, HCM = hypertrophic cardiomyopathy, HFH = heart failure hospitalization, HR = hazard ratio, LVEDD = left ventricular end‐diastolic dimension, LVESD = left ventricular end‐systolic dimension, MACCEs = major adverse cardiac and cerebrovascular events, Post‐LVOTV = postoperative left ventricular outflow tract velocity, Post‐SICU Vent Time = postoperative surgical intensive care unit ventilation time, SCD = sudden cardiac death, SOFA = sequential organ failure assessment, Surg Time = surgery time.

### 3.4 Kaplan−Meier Survival Analysis According to Multi‐Organ Dysfunction

**Figure 3:**
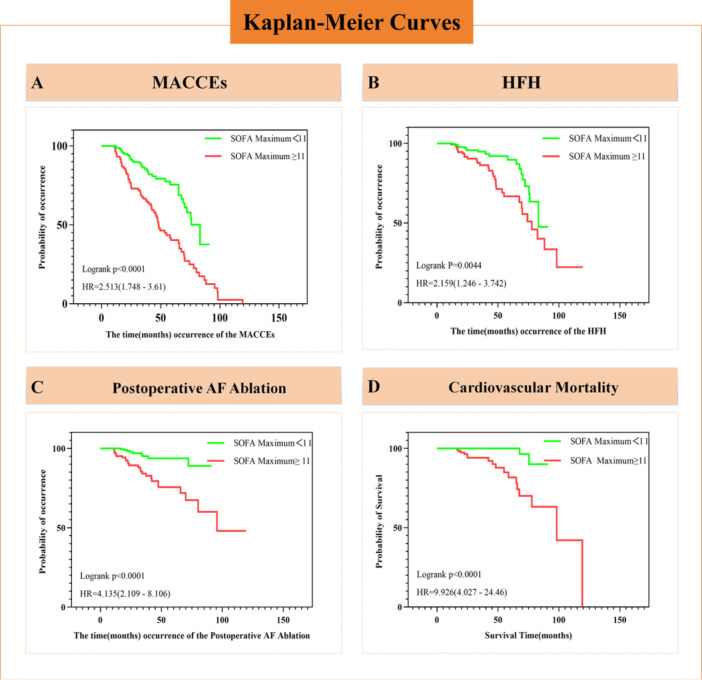
Primary Endpoint and Secondary Endpoints Kaplan−Meier Survival Curves. Figure 3(A, B, C, and D) show the survival curves for the end points MACCEs, HFH, postoperative AF ablation, and cardiovascular mortality, respectively. AF = atrial fibrillation, HFH = heart failure hospitalization, HR = hazard ratio, MACCEs = major adverse cardiac and cerebrovascular events, SOFA = Sequential Organ Failure Assessment.

This study also used Kaplan−Meier survival analysis to evaluate the impact of severe postoperative multi‐organ dysfunction on postoperative MACCEs, HFH, postoperative AF ablation, and cardiovascular mortality after modified Morrow surgery. The results demonstrated that patients with severe multi‐organ dysfunction had significantly higher risks for all endpoint events compared to those without multi‐organ severe dysfunction (Figure 3). The HR presented alongside the survival curves were unadjusted estimates derived from univariable Cox proportional hazards models. Specifically, the HR for MACCEs was 2.513 (95% CI: 1.748−3.61, log‐rank *p* < 0.0001, Figure 3A); for HFH it was 2.159 (95% CI: 1.246−3.742, log‐rank *p* = 0.0044, Figure 3B); for postoperative AF ablation it was 4.135 (95% CI: 2.109−8.106, log‐rank *p* < 0.0001, Figure 3C); and for cardiovascular mortality it was 9.926 (95% CI: 4.027−24.46, log‐rank *p* < 0.0001, Figure 3D). These findings indicate that severe multi‐organ dysfunction is associated with an increased risk of adverse cardiac and cerebrovascular events and poor survival outcomes following surgery.

## 4 Discussion

This study aimed to investigate the impact of severe postoperative multi‐organ dysfunction on long‐term outcomes in patients with HOCM undergoing the modified Morrow surgery. Through a retrospective cohort analysis of 380 patients, we found that individuals with a maximum SOFA score ≥ 11 had significantly higher rates of long‐term MACCEs. Specifically, severe multi‐organ dysfunction showed significant positive correlations with cardiovascular mortality, HFH, postoperative AF ablation, and the MACCEs composite endpoint. Multivariate analysis further confirmed that severe multi‐organ dysfunction was independently associated with these adverse outcomes. These results suggest that early postoperative multi‐organ functional status holds important predictive value for long‐term prognosis in HOCM patients.

In interpreting the composite MACCEs endpoint, we acknowledge that its individual components carry varying clinical significance. However, it is noteworthy that severe multi‐organ dysfunction demonstrated a consistent and independent association not only with the overall composite endpoint but also with highly critical individual components, such as cardiovascular mortality and HFH. This internal consistency across events of varying clinical significance suggests that the prognostic value of the SOFA score is robust and clinically relevant.

Our findings differ significantly from previous studies suggesting that severe multi‐organ dysfunction may be reversible with patient recovery [12]. We found that patients with multi‐organ dysfunction had significantly higher risks of MACCEs, HFH, postoperative AF ablation, and cardiovascular mortality during a median follow‐up of 35.9 months, suggesting that severe multi‐organ dysfunction is strongly associated with sustained systemic vulnerability rather than representing transient functional abnormalities. This finding aligns with the proposition by Lone et al. that organ failure may serve as a crucial clinical marker or contributing factor for long‐term adverse prognosis [13, 14]. This study, through large‐sample data and multivariate analysis, suggests that early severe multi‐organ dysfunction is independently associated with long‐term cardiovascular prognosis, providing new evidence to support the long‐term management of multi‐organ dysfunction after cardiac surgery.

The potential mechanisms by which severe postoperative multi‐organ dysfunction leads to long‐term adverse outcomes may involve multiple aspects. Previous studies have indicated that severe multi‐organ dysfunction is a significant contributor to morbidity and mortality following cardiac surgery, particularly in patients undergoing cardiopulmonary bypass (CPB) [19]. Additionally, Pölzl et al. demonstrated that myocardial injury is significantly associated with both 30‐day mortality and long‐term survival [20]. Our results showed that the severe multi‐organ dysfunction group had longer surgery times and post‐SICU Vent Time, leading to prolonged CPB times during surgery and frequently requiring more inotropic support postoperatively. These interventions themselves may cause secondary myocardial injury. Furthermore, dysregulated inflammatory response serves as a key driver of severe multi‐organ dysfunction, demonstrating significant pathway overlap with septic shock [19], while infected patients consistently exhibit higher SOFA scores compared to non‐infected counterparts [21]. Therefore, the persistent inflammatory state may activate the neuroendocrine system, promoting ventricular remodeling, myocardial injury, and electrophysiological instability, thereby increasing the risk of future heart failure and arrhythmias. Overall, severe multi‐organ dysfunction often arises from a complex interplay of intraoperative hypoperfusion, reperfusion injury, and systemic inflammatory response syndrome. These profound pathological stressors are hypothesized to be associated with cardiomyocyte apoptosis and microvascular dysfunction, potentially contributing to adverse ventricular remodeling and electrophysiological alterations. Such mechanisms may underline the potential association with the significantly elevated risk of long‐term cardiovascular events observed in our cohort.

Beyond severe multi‐organ dysfunction, our multivariate Cox regression analysis identified additional independent prognostic risk factors specific to HOCM patients undergoing modified Morrow surgery. Previous studies have identified family history of HCM as a risk factor for long‐term adverse outcomes following modified Morrow surgery [1, 4, 22, 23], which is consistent with our findings. Family history of HCM showed significant associations with cardiovascular mortality and HFH, suggesting that genetic background plays an important role in disease progression. Furthermore, structural and procedural variables, such as LVESD and Post‐SICU Vent Time, were also identified as independent predictors for MACCEs and cardiovascular mortality, respectively. These findings highlight the multifactorial nature of long‐term outcomes in this population, encompassing both genetic predisposition and perioperative clinical status.

This study carries important clinical implications. The SOFA score, as a widely used assessment tool in the Intensive Care Unit, can effectively identify patients at high risk for long‐term adverse outcomes. Given the observational nature of this study, the findings represent associations rather than established causal relationships. Consequently, our results do not directly support the adoption of more aggressive therapeutic interventions. Instead, the occurrence of severe postoperative multi‐organ dysfunction should serve as a critical clinical marker for identifying patients at highly elevated long‐term risk. For this vulnerable cohort, meticulous adherence to standard supportive care—including the optimization of organ perfusion, strict infection control, and close monitoring for arrhythmias—is paramount. Whether specific, intensified management strategies can effectively mitigate these long‐term cardiovascular risks remains to be determined by future prospective clinical trials. Furthermore, these patients should receive closer long‐term follow‐up, including regular cardiac function assessments and arrhythmia screening. Overall, severe multi‐organ dysfunction after cardiac surgery presents not only a challenge for acute management but is also a critical factor affecting patients’ long‐term quality of life and prognosis, requiring comprehensive intervention spanning preoperative risk assessment, intraoperative organ protection, and long‐term follow‐up [24].

The SOFA score has been validated as a risk stratification tool for cardiac surgery patients and certain cardiac conditions due to its simplicity, ease of use, and demonstrated effectiveness, with applications in sepsis, cardiogenic shock, infective endocarditis, and other critical cardiovascular scenarios [11, 25, 26, 27]. For risk stratification in HOCM, previous studies have primarily focused on preoperative indicators such as clinical symptoms, family history, and cardiac magnetic resonance imaging findings [1, 4, 28, 29, 30]. Recently, some studies have begun exploring the use of postoperative biochemical markers in HOCM to assess their impact on long‐term adverse outcomes [31]. However, due to the complex interplay of factors following cardiac surgery, single‐parameter approaches often prove insufficiently comprehensive. The severe multi‐organ dysfunction represented by a SOFA Maximum ≥ 11 serves as a comprehensive postoperative indicator (encompassing six distinct systems) [32]. This supports the potential integration of the SOFA score into the risk stratification system for HOCM patients undergoing modified Morrow surgery, thereby providing valuable guidance for developing individualized treatment and follow‐up strategies.

## 5 Limitations

Our study has several limitations that should be acknowledged. First, as a single‐center retrospective cohort study, it is susceptible to potential selection bias and unmeasured confounding factors despite our efforts to adjust for known covariates in the multivariate analysis. Second, the generalizability of our findings may be limited as all data were derived from a high‐volume tertiary care center in China; validation in multi‐center, international cohorts is necessary. Third, we were unable to account for variations in postoperative medical therapy (e.g., beta‐blockers, antiarrhythmic drugs) and patient compliance, which could influence long‐term outcomes. Fourth, the SOFA score was calculated based on clinical data during the Intensive Care Unit stay, and its dynamic changes were not analyzed in relation to the timing of organ recovery. Fifth, the absolute number of events for certain specific outcomes, particularly cardiovascular mortality, was relatively low. Consequently, the multivariable findings for these endpoints may be subject to wider CIs and should be interpreted cautiously. Finally, although the SOFA score demonstrated significant predictive value, its sensitivity for some secondary endpoints was low, suggesting that it should be used as part of a comprehensive assessment rather than as a standalone tool. Future prospective, multi‐center studies are warranted to confirm our findings and to explore targeted interventions for this high‐risk population.

## 6 Conclusions

This study suggests that severe multi‐organ dysfunction following the modified Morrow procedure is independently associated with an increased risk of long‐term adverse outcomes in patients, demonstrating significant associations with MACCEs, HFH, postoperative AF ablation, and cardiovascular mortality risk. Our findings not only further validate established risk factors for long‐term major adverse cardiovascular outcomes after HOCM surgery (such as family history of HCM), but also support the potential integration of the SOFA score into the risk stratification system for HOCM patients undergoing modified Morrow surgery. These results underscore the importance of perioperative organ function protection in cardiac surgery and provide a practical tool for identifying high‐risk patients. Future research should focus on developing targeted interventions to improve long‐term prognosis in patients with multi‐organ dysfunction.

## Author Contributions

W.T. and W.J. conceived and designed the study. W.T., T.L., and X.Y. acquired the data. W.T., W.J., and Z.K. analyzed and interpreted the data. W.T. drafted the manuscript. W.T., W.J., T.L., Z.K., D.R., and X.Y. critically revised the manuscript for important intellectual content. W.T. performed statistical analysis. W.J. provided administrative, technical, or material support. W.J. and D.R. supervised the study. All authors read and approved the final manuscript.

## Conflicts of Interest

The authors declare no conflicts of interest.

## Supporting information


Supporting File

## Data Availability

The data that support the findings of this study are available from the corresponding author, [Jiayang Wang], upon reasonable request.
