# Supplementary material for: Impact of Severe Postoperative Multi‐Organ Dysfunction on Long‐Term Outcomes After Modified Morrow Surgery
Source: Clin Cardiol. 2026 Aug 3;49(8):e70434. doi: 10.1002/clc.70434 (PMC13430974; doi:10.1002/clc.70434)
Supplement: Supplementary file 1 — Supporting File [file CLC-49-e70434-s001.docx]

**Univariate Cox Regression Analysis for MACCEs**

| **Variable** | **Univariate analysis HR** | **95% CI** | **P-value** |
| --- | --- | --- | --- |
| Male | 1.130 | 0.7854 to 1.636 | 0.511 |
| Age, years | 1.012 | 0.9959 to 1.029 | 0.148 |
| BMI, kg/m^2^ | 1.007 | 0.9932 to 1.016 | 0.242 |
| Family history of HCM | 1.501 | 1.026 to 2.172 | 0.**037** |
| Family history of SCD | 1.109 | 0.7071 to 1.684 | 0.641 |
| Unexplained Syncope | 1.074 | 0.7192 to 1.577 | 0.720 |
| Chest discomfort | 0.6429 | 0.3191 to 1.534 | 0.292 |
| Diabetes mellitus | 1.138 | 0.5750 to 2.028 | 0.691 |
| Hypertension | 1.097 | 0.7603 to 1.576 | 0.619 |
| Hypercholesterolemia | 1.492 | 0.8556 to 2.442 | 0.151 |
| Coronary Artery Disease | 0.9447 | 0.5432 to 1.544 | 0.829 |
| LAD, mm | 1.020 | 0.9915 to 1.049 | 0.164 |
| LVESD, mm | 1.041 | 1.001 to 1.082 | **0.047** |
| LVEDD, mm | 1.031 | 0.9941 to 1.069 | 0.101 |
| Moderate/Severe MR | 0.7761 | 0.5272 to 1.166 | 0.217 |
| LVOTG, mmHg | 0.9993 | 0.9948 to 1.004 | 0.753 |
| LVOTV, cm/s | 0.9996 | 0.9981 to 1.001 | 0.570 |
| SAM | 0.7729 | 0.4783 to 1.320 | 0.331 |
| Post-LVOTV, cm/s | 1.000 | 0.9971 to 1.003 | 0.990 |
| Post-LVOTG, mmHg | 0.9958 | 0.9830 to 1.004 | 0.346 |
| LVPWT, mm | 0.9645 | 0.9055 to 1.022 | 0.230 |
| IVST, mm | 0.9874 | 0.9517 to 1.023 | 0.485 |
| E/A | 1.069 | 0.7471 to 1.473 | 0.703 |
| LVEF, % | 0.9954 | 0.9687 to 1.023 | 0.739 |
| BNP, pg/mL | 1.000 | 0.9997 to 1.000 | 0.681 |
| CK-MB, ng/mL | 1.000 | 0.9888 to 1.006 | 0.995 |
| LDH, U/L | 0.9992 | 0.9962 to 1.001 | 0.416 |
| NSVT | 1.291 | 0.8487 to 1.918 | 0.227 |
| Surg Time, hours | 1.190 | 1.060 to 1.328 | **0.004** |
| Post-SICU Vent Time, hours | 1.003 | 1.001 to 1.004 | **0.008** |
| **Severe Multi-organ Dysfunction (**SOFA Maximum≥11) | 2.655 | 1.816 to 3.933 | **<0.001** |

MACCEs=Major Adverse Cardiac and Cerebrovascular Events; HR=Hazard Ratio; CI=confidence interval; BMI=body mass index; HCM=hypertrophic cardiomyopathy; SCD=sudden cardiac death; LAD=left atrial diameter; LVESD=left ventricular end-systolic dimension; LVEDD= left ventricular end-diastolic dimension; Moderate/Severe MR=moderate to severe mitral regurgitation; LVOTG=left ventricular outflow tract gradient; LVOTV=left ventricular outflow tract velocity; SAM=systolic anterior motion of the mitral valve; Post-LVOTV=Postoperative left ventricular outflow tract velocity; Post-LVOTG= postoperative left ventricular outflow tract gradient; LVPWT=left ventricular posterior wall thickness; IVST= interventricular septal thickness; E/A= early to late diastolic mitral inflow velocity ratio; LVEF= left ventricular ejection fraction; BNP= B-type natriuretic peptide; CK-MB= creatine kinase-myocardial band; LDH= lactate dehydrogenase; NSVT= non-sustained ventricular tachycardia; Surg Time=surgery time; Post-SICU Vent Time=postoperative surgical intensive care unit ventilation time; SOFA=sequential organ failure assessment.

**Univariate Cox Regression Analysis for HFH**

| **Variable** | **Univariate analysis HR** | **95% CI** | **P-value** |
| --- | --- | --- | --- |
| Male | 0.6963 | 0.3984 to 1.203 | 0.195 |
| Age, years | 1.021 | 0.9963 to 1.047 | 0.098 |
| BMI, kg/m^2^ | 1.012 | 0.9956 to 1.022 | 0.119 |
| Family history of HCM | 2.456 | 1.415 to 4.263 | **0.002** |
| Family history of SCD | 1.807 | 0.9811 to 3.196 | 0.057 |
| Unexplained Syncope | 1.435 | 0.8056 to 2.504 | 0.215 |
| Chest discomfort | 0.5745 | 0.2052 to 2.394 | 0.395 |
| Diabetes mellitus | 1.536 | 0.5846 to 3.356 | 0.352 |
| Hypertension | 0.8792 | 0.4973 to 1.524 | 0.649 |
| Hypercholesterolemia | 1.984 | 0.8992 to 3.910 | 0.086 |
| Coronary Artery Disease | 0.8009 | 0.3238 to 1.693 | 0.584 |
| LAD, mm | 0.9998 | 0.9547 to 1.044 | 0.992 |
| LVESD, mm | 1.003 | 0.9434 to 1.064 | 0.932 |
| LVEDD, mm | 0.9741 | 0.9220 to 1.029 | 0.348 |
| Moderate/Severe MR | 0.5609 | 0.3205 to 1.011 | 0.054 |
| LVOTG, mmHg | 1.002 | 0.9960 to 1.008 | 0.457 |
| LVOTV, cm/s | 1.001 | 0.9985 to 1.003 | 0.526 |
| SAM | 0.8198 | 0.4002 to 1.922 | 0.623 |
| Post-LVOTV, cm/s | 1.001 | 0.9970 to 1.006 | 0.533 |
| Post-LVOTG, mmHg | 0.9979 | 0.9823 to 1.006 | 0.697 |
| LVPWT, mm | 0.9500 | 0.8613 to 1.037 | 0.261 |
| IVST, mm | 1.009 | 0.9569 to 1.060 | 0.738 |
| E/A | 1.114 | 0.6493 to 1.763 | 0.676 |
| LVEF, % | 1.012 | 0.9713 to 1.056 | 0.565 |
| BNP, pg/mL | 1.000 | 0.9998 to 1.000 | 0.469 |
| CK-MB, ng/mL | 1.004 | 0.9934 to 1.010 | 0.370 |
| LDH, U/L | 1.000 | 0.9971 to 1.002 | 0.884 |
| NSVT | 1.527 | 0.8269 to 2.708 | 0.170 |
| Surg Time, hours | 1.039 | 0.8571 to 1.243 | 0.691 |
| Post-SICU Vent Time, hours | 1.002 | 0.9989 to 1.005 | 0.163 |
| **Severe Multi-organ Dysfunction (**SOFA Maximum≥11) | 2.231 | 1.274 to 3.991 | **0.005** |

HFH=Heart Failure Hospitalization; HR=Hazard Ratio; CI=confidence interval; BMI=body mass index; HCM=hypertrophic cardiomyopathy; SCD=sudden cardiac death; LAD=left atrial diameter; LVESD=left ventricular end-systolic dimension; LVEDD= left ventricular end-diastolic dimension; Moderate/Severe MR=moderate to severe mitral regurgitation; LVOTG=left ventricular outflow tract gradient; LVOTV=left ventricular outflow tract velocity; SAM=systolic anterior motion of the mitral valve; Post-LVOTV=Postoperative left ventricular outflow tract velocity; Post-LVOTG= postoperative left ventricular outflow tract gradient; LVPWT=left ventricular posterior wall thickness; IVST= interventricular septal thickness; E/A= early to late diastolic mitral inflow velocity ratio; LVEF= left ventricular ejection fraction; BNP= B-type natriuretic peptide; CK-MB= creatine kinase-myocardial band; LDH= lactate dehydrogenase; NSVT= non-sustained ventricular tachycardia; Surg Time=surgery time; Post-SICU Vent Time=postoperative surgical intensive care unit ventilation time; SOFA=sequential organ failure assessment.

**Univariate Cox Regression Analysis** for **Postoperative AF Ablation**

| **Variable** | **Univariate analysis HR** | **95% CI** | **P-value** |
| --- | --- | --- | --- |
| Male | 1.677 | 0.8490 to 3.489 | 0.138 |
| Age, years | 1.014 | 0.9858 to 1.046 | 0.334 |
| BMI, kg/m^2^ | 0.9951 | 0.9168 to 1.017 | 0.807 |
| Family history of HCM | 1.574 | 0.7816 to 3.076 | 0.198 |
| Family history of SCD | 1.705 | 0.8003 to 3.407 | 0.159 |
| Unexplained Syncope | 1.765 | 0.8833 to 3.450 | 0.106 |
| Chest discomfort | 1.710 | 0.3661 to 30.46 | 0.565 |
| Diabetes mellitus | 0.2942 | 0.01655 to 1.365 | 0.139 |
| Hypertension | 1.097 | 0.5553 to 2.137 | 0.787 |
| Hypercholesterolemia | 1.878 | 0.7016 to 4.237 | 0.192 |
| Coronary Artery Disease | 0.7578 | 0.2238 to 1.932 | 0.592 |
| LAD, mm | 1.010 | 0.9570 to 1.061 | 0.702 |
| LVESD, mm | 1.054 | 0.9797 to 1.132 | 0.158 |
| LVEDD, mm | 1.047 | 0.9799 to 1.120 | 0.175 |
| Moderate/Severe MR | 0.7414 | 0.3710 to 1.579 | 0.422 |
| LVOTG, mmHg | 0.9970 | 0.9880 to 1.005 | 0.483 |
| LVOTV, cm/s | 0.9984 | 0.9956 to 1.001 | 0.247 |
| SAM | 0.6585 | 0.2896 to 1.777 | 0.380 |
| Post-LVOTV, cm/s | 0.9939 | 0.9881 to 0.9995 | **0.033** |
| Post-LVOTG, mmHg | 0.9820 | 0.9442 to 1.005 | 0.204 |
| LVPWT, mm | 0.9697 | 0.8614 to 1.074 | 0.576 |
| IVST, mm | 0.9341 | 0.8694 to 1.001 | 0.055 |
| E/A | 1.343 | 0.7354 to 2.204 | 0.314 |
| LVEF, % | 0.9835 | 0.9376 to 1.033 | 0.504 |
| BNP, pg/mL | 1.000 | 0.9995 to 1.000 | 0.987 |
| CK-MB, ng/mL | 0.9294 | 0.7276 to 1.006 | 0.211 |
| LDH, U/L | 0.9980 | 0.9905 to 1.002 | 0.432 |
| NSVT | 1.561 | 0.7331 to 3.118 | 0.237 |
| Surg Time, hours | 1.269 | 1.036 to 1.527 | **0.022** |
| Post-SICU Vent Time, hours | 1.002 | 0.9981 to 1.005 | 0.265 |
| **Severe Multi-organ Dysfunction (**SOFA Maximum≥11) | 4.318 | 2.086 to 9.799 | **<0.001** |

AF=atrial fibrillation; HR=Hazard Ratio; CI=confidence interval; BMI=body mass index; HCM=hypertrophic cardiomyopathy; SCD=sudden cardiac death; LAD=left atrial diameter; LVESD=left ventricular end-systolic dimension; LVEDD= left ventricular end-diastolic dimension; Moderate/Severe MR=moderate to severe mitral regurgitation; LVOTG=left ventricular outflow tract gradient; LVOTV=left ventricular outflow tract velocity; SAM=systolic anterior motion of the mitral valve; Post-LVOTV=Postoperative left ventricular outflow tract velocity; Post-LVOTG= postoperative left ventricular outflow tract gradient; LVPWT=left ventricular posterior wall thickness; IVST= interventricular septal thickness; E/A= early to late diastolic mitral inflow velocity ratio; LVEF= left ventricular ejection fraction; BNP= B-type natriuretic peptide; CK-MB= creatine kinase-myocardial band; LDH= lactate dehydrogenase; NSVT= non-sustained ventricular tachycardia; Surg Time=surgery time; Post-SICU Vent Time=postoperative surgical intensive care unit ventilation time; SOFA=sequential organ failure assessment.

**Univariate Cox Regression Analysis for Cardiovascular Mortality**

| **Variable** | **Univariate analysis HR** | **95% CI** | **P-value** |
| --- | --- | --- | --- |
| Male | 0.4531 | 0.1573 to 1.172 | 0.104 |
| Age, years | 1.065 | 1.017 to 1.121 | **0.006** |
| BMI, kg/m^2^ | 1.018 | 0.9969 to 1.030 | 0.080 |
| Family history of HCM | 12.23 | 3.997 to 53.05 | **<0.001** |
| Family history of SCD | 2.860 | 1.064 to 7.385 | **0.038** |
| Unexplained Syncope | 1.701 | 0.6397 to 4.362 | 0.277 |
| Chest discomfort | 0.4592 | 0.08509 to 8.500 | 0.506 |
| Diabetes mellitus | 0.7304 | 0.04040 to 3.605 | 0.750 |
| Hypertension | 0.8415 | 0.3087 to 2.144 | 0.720 |
| Hypercholesterolemia | 1.760 | 0.3984 to 5.491 | 0.411 |
| Coronary Artery Disease | 1.342 | 0.3593 to 3.920 | 0.630 |
| LAD, mm | 0.9871 | 0.9083 to 1.064 | 0.747 |
| LVESD, mm | 0.9337 | 0.8365 to 1.036 | 0.198 |
| LVEDD, mm | 0.8960 | 0.8112 to 0.9852 | **0.023** |
| Moderate/Severe MR | 0.5227 | 0.2018 to 1.457 | 0.204 |
| LVOTG, mmHg | 1.003 | 0.9928 to 1.013 | 0.512 |
| LVOTV, cm/s | 1.001 | 0.9973 to 1.005 | 0.605 |
| SAM | 0.5739 | 0.1953 to 2.173 | 0.379 |
| Post-LVOTV, cm/s | 0.9987 | 0.9911 to 1.006 | 0.727 |
| Post-LVOTG, mmHg | 0.9913 | 0.9446 to 1.009 | 0.506 |
| LVPWT, mm | 0.9636 | 0.8136 to 1.107 | 0.627 |
| IVST, mm | 1.047 | 0.9639 to 1.129 | 0.262 |
| E/A | 1.223 | 0.4628 to 2.567 | 0.652 |
| LVEF, % | 1.017 | 0.9470 to 1.095 | 0.652 |
| BNP, pg/mL | 1.000 | 0.9994 to 1.001 | 0.823 |
| CK-MB, ng/mL | 1.009 | 0.9986 to 1.015 | 0.079 |
| LDH, U/L | 0.9998 | 0.9928 to 1.002 | 0.925 |
| NSVT | 2.554 | 0.9536 to 6.578 | 0.061 |
| Surg Time, hours | 1.352 | 1.019 to 1.717 | **0.037** |
| Post-SICU Vent Time, hours | 1.006 | 1.003 to 1.008 | **0.001** |
| **Severe Multi-organ Dysfunction (**SOFA Maximum≥11) | 11.42 | 3.219 to 72.51 | **<0.001** |

HR=Hazard Ratio; CI=confidence interval; BMI=body mass index; HCM=hypertrophic cardiomyopathy; SCD=sudden cardiac death; LAD=left atrial diameter; LVESD=left ventricular end-systolic dimension; LVEDD= left ventricular end-diastolic dimension; Moderate/Severe MR=moderate to severe mitral regurgitation; LVOTG=left ventricular outflow tract gradient; LVOTV=left ventricular outflow tract velocity; SAM=systolic anterior motion of the mitral valve; Post-LVOTV=Postoperative left ventricular outflow tract velocity; Post-LVOTG= postoperative left ventricular outflow tract gradient; LVPWT=left ventricular posterior wall thickness; IVST= interventricular septal thickness; E/A= early to late diastolic mitral inflow velocity ratio; LVEF= left ventricular ejection fraction; BNP= B-type natriuretic peptide; CK-MB= creatine kinase-myocardial band; LDH= lactate dehydrogenase; NSVT= non-sustained ventricular tachycardia; Surg Time=surgery time; Post-SICU Vent Time=postoperative surgical intensive care unit ventilation time; SOFA=sequential organ failure assessment.
